# 

**Published:** 1891-09

**Authors:** 


					﻿ESTABLISHED 1S55
SUBSCRIPTiONZSl

II. V. M. MILLER, M. D., LL. D.
VIRGIL O. HARDON, M. D.
F. W. McRAE, M. D.
L.	I’. KENNEDY, A M . M. 1).
M.	B. Ill TCIIINS, M I).
L. B. GRANDY, M. D.
l’rni.i'HKi) by JAS. I’. HARRISON A co.. Ati.o r>r,'<a.
Entered at the P<»st-< tfiice at Atlanta, Ga.. as	InatTei	j
SwSZtV^m ATLANTA, G.A., SePTEMBeL is/t*/' No . 1
				

